# Molecular Pathogenesis of Neuromyelitis Optica

**DOI:** 10.3390/ijms131012970

**Published:** 2012-10-11

**Authors:** Wajih Bukhari, Michael H Barnett, Kerri Prain, Simon A Broadley

**Affiliations:** 1 School of Medicine, Gold Coast Campus, Griffith University, QLD 4222, Australia; E-Mail: simon.broadley@griffith.edu.au; 2 Department of Neurology, Gold Coast Hospital, Southport, QLD 4215, Australia; 3 Brain and Mind Research Institute, Camperdown, NSW 2050, Australia; E-Mail: michael@sydneyneurology.com.au; 4 Autoimmune laboratory, Division of Immunology, Pathology Queensland, Herston, QLD 4029, Australia; E-Mail: kerri_prain@health.qld.gov.au

**Keywords:** pathogenesis, Devic’s disease, immunology, genetics, neuromyelitis optica, multiple sclerosis, aquaporin-4, astrocytopathy, astrocyte

## Abstract

Neuromyelitis optica (NMO) is a rare autoimmune disorder, distinct from multiple sclerosis, causing inflammatory lesions in the optic nerves and spinal cord. An autoantibody (NMO IgG) against aquaporin-4 (AQP4), a water channel expressed on astrocytes is thought to be causative. Peripheral production of the antibody is triggered by an unknown process in genetically susceptible individuals. Anti-AQP4 antibody enters the central nervous system (CNS) when the blood brain barrier is made permeable and has high affinity for orthogonal array particles of AQP4. Like other autoimmune diseases, Th17 cells and their effector cytokines (such as interleukin 6) have been implicated in pathogenesis. AQP4 expressing peripheral organs are not affected by NMO IgG, but the antibody causes extensive astrocytic loss in specific regions of the CNS through complement mediated cytotoxicity. Demyelination occurs during the inflammatory process and is probably secondary to oligodendrocyte apoptosis subsequent to loss of trophic support from astrocytes. Ultimately, extensive axonal injury leads to severe disability. Despite rapid advances in the understanding of NMO pathogenesis, unanswered questions remain, particularly with regards to disease mechanisms in NMO IgG seronegative cases. Increasing knowledge of the molecular pathology is leading to improved treatment strategies.

## 1. Introduction

A syndrome of severe demyelination affecting the optic nerves and spinal cord specifically was described in the mid-late 19th century by Allbutt and Erb [1,2] and possibly even earlier by others [3]. More detailed phenotypic features, including simultaneous, sequential and relapsing-remitting forms of the ocular and spinal manifestations together with detailed pathological studies were provided by Devic [4]. Whilst further phenotypic clues emerged over the following century, including cerebrospinal fluid (CSF) pleiocytosis, elevated CSF protein and normal magnetic resonance imaging (MRI) brain [5,6], the clinical description of neuromyelitis optica (NMO) and the potential overlap with MS remained essentially unchanged until the discovery of a specific antibody (NMO IgG) in 2004 [7].

The clinical features of NMO are compared with MS in Table 1. NMO is characterised clinically by synchronous or sequential optic neuritis and longitudinally extensive spinal cord inflammation. Monophasic and relapsing courses are recognised; progressive disease is uncommon [8,9]. NMO is typically more severe than MS and is more likely to result in significant residual loss of vision and immobility following attacks [6,10]. MRI of the brain is typically normal initially and the CSF shows elevated protein and a lymphocytic pleiocytosis [6]. Oligoclonal bands are less commonly seen in NMO than in MS [11]. Since the discovery of NMO IgG the phenotype associated with NMO has broadened to include an encephalopathic presentation (sometimes with large diffuse cerebral white matter lesions) [12–14], recurrent optic neuritis or cord disease (including partial cord lesions) [14,15], intractable hiccups (particularly in childhood) [16,17] and an acute brain stem syndrome [17]. In addition to optic nerve involvement with or without nonspecific white matter lesions [6,13], other abnormalities found on brain MRI include lesions of the hypothalamus [12], periaqueductal grey matter [12,18] and splenium of the corpus collosum [19]. Confluent periventricular lesions are also rarely observed, mostly in fulminant cases [20,21]. The gender ratio for NMO is much higher (female:male = 9:1) [10] than it is for MS (3:1) [22]. An association with other autoimmune diseases has been frequently reported [23], in contrast with MS, which either does not show any association [24,25] or only a mild association with systemic autoimmunity [26,27].

Formal diagnostic criteria have been established for NMO [13]. Whilst a number of different diagnostic criteria have been proposed the most widely accepted require the presence of optic neuritis, acute myelitis and at least two out of: (1) contiguous spinal cord MRI lesion extending over ≥3 vertebral segments; (2) brain MRI not meeting diagnostic criteria for multiple sclerosis; and (3) NMO IgG seropositive status [13]. Using these criteria, NMO IgG is found to be highly specific for NMO. However, these criteria do not capture a large number of cases that have an incomplete clinical picture. This includes cases of isolated or recurrent optic neuritis, isolated myelitis and more recently cases of recurrent hiccoughs in childhood, an acute brainstem syndrome, encephalitis involving the white matter and other unusual presentations which have been associated with NMO IgG and have been collectively labelled as NMO spectrum disorder [14].

As a seemingly antigen specific autoimmune disease with potentially dire consequences, treatment approaches in NMO are generally more aggressive than in MS and usually incorporates immunosuppressive therapy. There is increasing evidence that conventional treatment for MS, in the form of β-interferon, may be associated with a worse outcome in NMO [28–30]. Initial treatment of attacks in NMO is usually with high dose intravenous corticosteroids [6] or if this fails plasmapheresis [31]. Maintenance treatment with oral corticosteroids and immunosuppression (cyclophosphamide, azathioprine, mycophenolate mofetil, methotrexate) is recommended as first line treatment [32]. There is growing evidence for the role of rituximab therapy in NMO [33,34]. This treatment approach makes logical sense given the presumed pathogenic role of NMO IgG.

The discovery of NMO IgG, which is directed against aquaporin-4 (AQP4), has dramatically changed the clinical definition of NMO and our understanding of the pathophysiology of this disease. A picture is finally emerging of NMO as a distinct pathological entity rather than a variant of MS, although some areas of confusion remain.

In NMO, involvement of the optic nerves and the central grey matter of the spinal cord is common and may lead to severe loss of vision, disability and even death [6]. The brain is often relatively spared in the early stages of the disease [6]. The central pathological process in NMO is the production of abnormal auto-antibodies against AQP4 expressed on astrocytes. These autoantibodies mediate complement dependent necrosis of astrocytes [35] and subsequent demyelination [36] together with significant loss of neurons [37]. The aim of this review is to summarise the roles played by key molecules in the pathogenesis of NMO. The nexus between the molecular basis of NMO and potential therapeutic approaches will be explored.

## 2. Pathogenesis of NMO

### 2.1. Aquaporin 4

Aquaporins are water channels present on cell membranes [38]. Of 13 different aquaporins, AQP4 is the predominant type found in mammalian brain [39]. The AQP4 gene is located on chromosome 18. The gene product is a bidirectional, osmosis driven water channel mainly expressed on the foot processes of astrocytes and on ependymal cells in the CNS [40]. It is also expressed in many other organs such as the kidneys, and the gastrointestinal and respiratory tracts [41]. In the CNS AQP4 is preferentially expressed in the retina [42], optic nerve, hypothalamus, cerebellum, periventricular and periaqueductal regions, and the spinal cord [40], and low levels are observed in the cerebral cortex [40,43]. AQP4 forms as a tetramer [44] and each AQP4 monomer has two splicing isoforms identified as M1 and M23. M1 (32 kDa) is 22 amino acids longer than M23 (30 kDa) which is spliced in a shorter formation at the *N*-terminus [45]. M23 is the predominant isoform in the CNS [46]. AQP4 tetramers are arranged in groups in the form of orthogonal arrays of particles (OAP) on the cell membrane. The size and conformation of these arrays is determined by the relative amount of M23 *versus* M1 present in the tetramers [47]. AQP4 OAPs have been likened to rafts. The M1 isoform limits the size of OAPs but M23 facilitates formation of larger aggregates [48]. Post translationally, palmitic acid binds with *N*-terminal cysteines of M1 and inhibits orthogonal array formation [49]. AQP4 is associated with the glutamate transporter, excitatory amino acid transporter 2 (EAAT2). In AQP4-transfected cells, EAAT2 is internalised during AQP4 endocytosis [50,51], a process that has not been observed in primary astrocyte cultures or *in vivo* experiments [52].

AQP4 knockout mice do not show any neurological deficits in health [53] but show altered response in disease states. For example, AQP4 knockout mice show reduced cytotoxic oedema of the brain in stroke [53], reduced glial scar formation [54], increased vasogenic oedema with brain tumours [55] and CNS infection [56], and a more severe form of induced hydrocephalus [56].

### 2.2. NMO IgG

**Figure 1:**
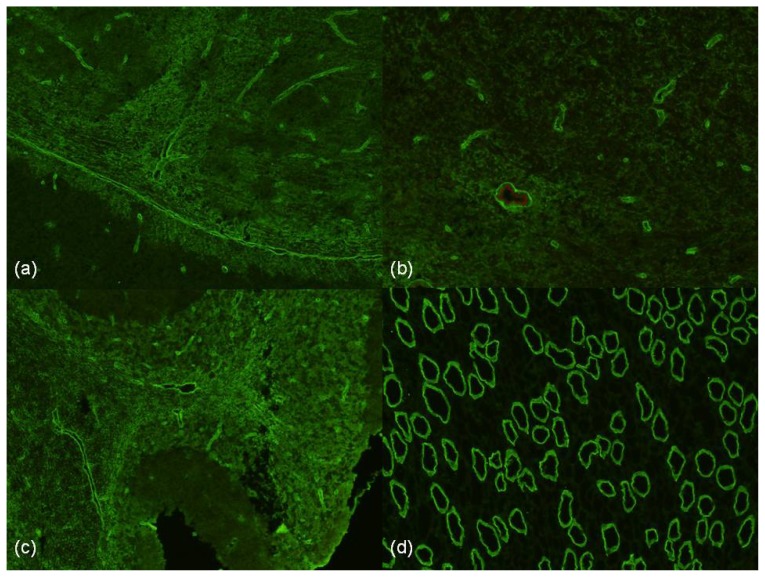
NMO positive Immunofluorescence on a composite of mouse cerebellum, midbrain and kidney (serum dilution 1:40, goat anti-human IgG F(ab)2 fluorescein isothiocyanate, 200× magnification). (**a**) and (**b**) staining of subpial surface and microvessels of the midbrain. (**c**) Microvessel staining of cerebellar granular layer, molecular layer and white matter. (**d**) Staining of the papillary tubules of the kidney.

Antibodies against AQP4, originally identified as NMO IgG, were first demonstrated through standard immunofluorescence techniques using various substrates, including mouse brain and kidney [7]. Classical staining of the subpial surface, microvessels of brain and cerebellum and papillary tubules of the kidney is illustrated in Figure 1. Subsequently enzyme linked immunosorbent assay (ELISA) and live cell-based assays have been developed with live cell-based assays utilising the M23 isoform of AQP4 having the highest sensitivity [57].

NMO IgG has high specificity (99%) [58,59] and moderate sensitivity ranging from 56% [58] to 73% [7,58] for NMO. It has been observed that the sensitivity of the autoantibody is higher in relapsing cases of NMO [58]. The autoantibody in the blood of NMO patients is predominantly the IgG1 isotype (98% of cases) [60], which can potently activate the complement system [61]. IgG2, IgG3 and IgG4 also occur in a lower proportion of cases [60]. IgM NMO antibody has also been reported in the blood of up to 10% of patients with NMO but it is not known to exist in the absence of IgG [62]. NMO IgG binds to the third extracellular loop of AQP4 [63] and the generation of conformational epitopes during OAP formation results in preferential binding with the M23 isoform [64]. One study has suggested that NMO IgG has considerably lower affinity for the AQP4 protein when compared with the epitope presented by OAPs [65]. NMO IgG does not cross the blood brain barrier (BBB) in normal subjects [66] but it can cross the placenta [67]. It has been demonstrated that NMO IgG is synthesised outside of the CNS. Persistent intrathecal synthesis of oligoclonal IgG, the most stable laboratory feature of MS, was absent in a study of 89 seropositive patients with NMO spectrum disorder, although transient intrathecal production was occasionally observed during acute relapses [11]. CSF from 20 NMO patients showed lower titres of NMO IgG in CSF than in serum (with a ratio of 1:500) in keeping with extrathecal synthesis of the autoantibody [68]. A further seven cases of NMO with low CSF antibody index of NMO IgG (AQP4 IgG/Total IgG) have since been reported [69]. In order to cause disease in the CNS, the extrathecally produced autoantibody requires disruption of the BBB (possible mechanisms for this are discussed below). NMO IgG is occasionally restricted to the CSF [70] and AQP4 specific B cells have been identified in the CSF of one patient with NMO [71]. In some patients, NMO IgG is produced by a subset of CD20 negative B cells (CD19^intermediate^CD27^high^CD38^high^CD180^−^) that resembled early plasma cells [72].

### 2.3. Triggers for Autoimmunity

A genetic predisposing factor is likely in NMO, as the disease is relatively more prevalent in non-Caucasian populations, as compared to MS which is more common in Europeans [73–76]. However, the stand alone prevalence of NMO (not relative to MS) has not been systematically determined in any large population. Further clues favouring a genetic predisposition derive from familial NMO cases. While such cases have been described for many years [77], only a small proportion (3%) of cases have a positive family history, predominantly those with Asian or Latino descent [78]. In the most comprehensive familial study to date (12 families, 25 affected individuals), the pattern of inheritance was thought to be complex [78]. The human leukocyte antigen (HLA) associations of NMO are contrasted with MS in Table 2. NMO IgG positivity has been found to be associated with HLA-DRB1*03 (DR3) in French (OR 3.08; 95% CI 1.52–6.27, *p* = 0.001) [79] and Brazilian (OR 3.23; 95% CI 1.07–9.82, *p* = 0.04) populations [80] and HLA-DPB1*0501 in Japanese (OR 4.8; 95% CI 1.6–14.3, *p* = 0.032) [81] and Chinese (OR 7.096; 95% CI 2.011–25.044, *p* = 0.022) populations [82]. Notably, these HLA haplotypes are also associated with other humoral autoimmune disorders such as systemic lupus erythematosus (SLE) [83] and Graves’ disease [84]. Indeed, NMO often coexists with other autoimmune conditions such as SLE and Sjögren syndrome [23] and these disorders are also more common in family members of patients with NMO [78]. Recent diagnostic criteria for NMO have tended to exclude SLE and Sjögren syndrome as not being compatible with a diagnosis of NMO [13].

Like many other autoimmune disorders NMO is also more common in women [10,88] suggesting that gender differences play a role in the pathogenesis of NMO. Genetic analysis of the *AQP4* gene was carried out to determine if mutations in the gene are associated with NMO [89]. No significant association was found with the 8 selected SNPs. However, at position number 3180 in the *AQP4* gene, which transcripts arginine at position 19, two missense mutations were observed in 3 out of 191 cases of NMO but not in any of 1363 controls. Whilst not statistically significant, it was speculated that loss of arginine 19 may impair palmitoylation of AQP4, which may increase the size of OAPs [49] and thereby generate immunogenic epitopes. Since Arginine 19 is in the regulatory sequence of M23 [90], this mutation could result in dysregulation of the transcription or translation of M23, resulting in a potentially immunogenic conformational change in OAPs. As described previously NMO IgG has high affinity for epitopes expressed in OAPs but lower affinity for isolated AQP4 protein [65].

In one series the onset of neurological symptoms in almost 30% of NMO cases was preceded by a viral or bacterial infection (more cases associated with varicella zoster and mycobacterium tuberculosis) [91] and vaccination against human papilloma virus has been reported to precede the onset of NMO in 4 teenage girls [92]. Parainfectious NMO may have a different immunopathogenesis as the majority of these cases have a monophasic course [91]. Rarely, a paraneoplastic association has also been described in NMO, especially with carcinoma of the breast and lung, and B cell-lymphoma [93]. A recent study of the T cells derived from the blood of 15 NMO patients has supported molecular mimicry as a possible cause of autoimmunity in NMO. Homology between the immunogenic amino acids 66–75 of *AQP4* and the surface protein ABC-TP of clostridia species especially *Clostridium perfringens* has been observed and ABC-TP was shown to cross react with anti-AQP4 T cells [94]. *Clostridium perfringens* is a ubiquitous organism and can occur in human gut as a commensal [95]. This study requires independent replication.

In MS, autoantibodies against myelin and other CNS antigens have been variably reported in as many as 60%–70% of cases [96] and the levels of autoantibodies fluctuate with relapses of disease [97]. However, no consistent high affinity serum autoantibody response is observed in MS and B-cells may be sensitised against self-antigens as an epiphenomenon during relapses. Similarly, there is a possibility that an initial disease process in the CNS of NMO patients attracts B-cells and sensitises them against AQP4. However, in an experiment to detect spontaneous production of NMO IgG in murine experimental autoimmune encephalomyelitis (EAE), extensive infiltration of immune cells into the CNS was seen without any detectable NMO IgG [98].

It remains to be discovered what, if any, role is played by molecular mimicry, bystander activation or a persistent infection in the initiation of autoantibody production in NMO.

### 2.4. Is Anti-AQP4 Antibody Pathogenic?

The antibody from the serum of NMO patients has been shown to exacerbate the clinical and pathological features of murine EAE [66,99] and to cause disease when injected intracerebrally in wild-type mice [35]. However, NMO IgG is only pathogenic in the presence of human complement proteins [35,100] or a pre-existing inflammatory state in the CNS such as occurs in EAE or mice pre-treated with Freund’s complete adjuvant [101]. The stronger the pre-existing CNS inflammation, the more severe the disease. Therefore, in EAE models more extensive disease is induced by NMO IgG [66,99], whereas only mild disease is observed in mice pre-treated with Freund’s complete adjuvant, which causes milder CNS inflammation than EAE [101]. AQP4 is expressed in non-CNS tissues [68,102], but no pathological involvement of extra neuronal tissues has been observed in NMO cases. This suggests that pathogenic potential of the autoantibody is restricted to the CNS. NMO IgG fails to produce disease in juvenile mice with incomplete BBB [66]; while access of NMO IgG to the CNS is a pre-requisite for the induction of disease, it is not sufficient to cause clinically relevant pathology in isolation. Higher titres of NMO IgG have been reported in patients with increased disease activity and severity [68,103], consistent with antibody pathogenicity. However, antibody titres do not correlate closely with disease activity in all studies [60,104,105]. More questions have been raised about the pathogenicity of NMO IgG following a case report in which NMO IgG was detected in blood 10 years before the clinical onset of the disease [106] and in another case series where NMO IgG was detected in 2 patients with cancer but without any clinical manifestation of NMO [93]. It can be concluded that NMO IgG is pathogenic, but only in a specific CNS milieu.

### 2.5. Immune Mediated Astrocytopathy

NMO IgG binds with AQP4 and activates complement making the lytic complex C5b-9, which destroys astrocytes [107]. Neutrophils, natural killer cells, macrophages, tumour necrosis factor α, interleukin (IL)-6, IL-1β, and interferon-gamma have been shown to amplify this complement mediated damage [100]. Complement regulatory proteins (CRP) on astrocytes such as decay-accelerating factor (CD55), membrane cofactor protein (CD46), complement receptor 1 and protectin (CD59) may also be dysfunctional in NMO, thus facilitating complement mediated astrocyte injury. In mice injected intracerebrally with NMO IgG. NMO-like lesions are only produced when human, but not mouse, complement is co-administered [35]. It is known that murine CRP may not have a potent protective effect against heterologous complement [108,109]. CRP dysfunction secondary to autoantibodies has been reported in MS [110]. The possibility of dysfunctional CRP in NMO requires further investigation.

Anti-AQP4 antibody has been reported in some studies to cause internalisation of AQP4 [50,61,111]. However, internalisation of AQP4, a process that could paradoxically protect astrocytes from immune mediated cytotoxicity, is unlikely to be the principal pathogenic mechanism in NMO. As noted previously the absence of AQP4 function does not result in neurological disorder in AQP4 knockout mice [53]. Additionally, little or no internalisation of antibody-AQP4 complex is observed in primary astrocyte culture or *in vivo*, emphasising the limited applicability of AQP4-transfected cells when modelling human disease [52,112]. It has also been shown that astrocytes do not suffer any osmotic injury as NMO IgG does not disturb the function of AQP4 [113,114].

Perivascular glial fibrillary acid protein (GFAP) positive astrocytes are consistently lost in NMO lesions in contrast with MS [36,37], where lesional GFAP is usually upregulated [115]. CSF levels of GFAP are markedly elevated during relapses of NMO but not MS [116]. GFAP is a cytoskeletal protein that plays a role in astrocyte motility and structure [115]. It is highly expressed by reactive astrocytes [117].

### 2.6. Immunopathogenesis

Systemic production of autoantibody with disease limited to the CNS implicates the presence of orchestrating T helper cells as well as a cascade of cytokines in the periphery and within the CNS. B cells are also attracted to the CNS by chemokines secreted at sites of inflammation. A study involving 31 NMO patients found various Th2 related cytokines such as IL-1 receptor antagonist, IL-5, IL-10 and IL-13; and the Th17 related cytokines IL-6, IL-8 and granulocyte colony stimulating factor to be elevated in the CSF. However, the signature Th2 cytokine, IL-4, and Th17 cytokine, IL-17, were not detected in the CSF [118]. Other studies have shown increased levels of IL-17 in the CSF of NMO patients [119,120]. Therefore, Th17 cells may have an intermittent role in the CNS inflammation that characterises NMO. Chitinases, hydrolases secreted by the innate immune cells that play a role in Th2 as well as Th1 related inflammatory processes, are also increased in the CSF of NMO patients. In response to IL-13 monocytes obtained from NMO patients secrete chitinases that increase *in vitro* chemotaxis of eosinophils, macrophages and T cells across the BBB by promoting the secretion of various cytokines IL-8, RANTES (CCL5), MCP1 (CCL2), and eotaxins by monocytes [121]. Eosinophils and macrophages are abundant in NMO lesions [122]. CSF levels of CXCL13, a potent chemo-attractant for B cells, are elevated and correlate with disease severity [123]. As indicated previously, anti-AQP4 B cells have been detected in the CSF of NMO patients [71]. CXCL13 is secreted by sensitised T helper cells [124] and antigen presenting cells (APC) [125]. B-cell activating factor (BAFF) is increased in the CSF but not serum of NMO patients [126]. BAFF, secreted by innate immune cells, APC [127] and activated astrocytes [128], increases the survival and maturation of B cells through NF-κB [129]. Thus, BAFF can perpetuate the auto-reactive B cell population in the CNS. It is interesting to note that treatment with both β-interferon and glatiramer acetate was found to increase levels of BAFF in the serum of MS patients [126]. This may account for the apparent worsening of NMO with β-interferon [28–30]. Despite similar changes in BAFF occurring with glatiramer acetate the same concerns regarding its use in NMO have not been raised [130,131].

IL-6 is elevated not only in the CSF but also in the serum of patients with NMO [118,132], particularly in patients with more severe disease [132]. This proinflammatory cytokine, which can also be secreted by activated astrocytes [133], increases the survival of NMO IgG producing B cells and the production of antibody [72]. In *ex vivo* experiments in murine spinal cord, IL-6 increased the induced NMO-like lesions [100]. In a study of 18 NMO patients, IL-9, a T cell growth factor, was elevated in the serum [134] but, not the CSF [118]. IL-6 is involved in the development of IL-17-secreting CD4^+^ T cells (Th17) [135]. Th17 cells have been implicated in many autoimmune disorders such as rheumatoid arthritis, psoriasis, MS and inflammatory bowel disease [136]. Th17 cells and their principal product, IL-17, are found to be increased in serum from patients with NMO [137,138], and some studies as seen above have also shown elevated IL-17 in the CSF [119,120], suggesting the involvement of Th17 cells and IL-17 in the pathogenesis of the disease. IL-17 is seen to mediate inflammation in the brain of EAE mice by promoting microglial activation [139]. Th17 cells may dictate the autoimmune milieu that facilitates the pathogenesis of NMO. A subset of B cells (CD27^high^CD38^high^CD180^−^ with low CD20 and CD19^−^), which may be producing anti-AQP4 antibody, appear to be activated [72]. Further implicating a role for humoral immunity in NMO, CD27^+^ memory B cell levels rise in association with relapses [34]. There is some evidence that cellular autoimmunity is also initiated as CD4^+^ T cells against AQP4 are detectable in the blood of patients with NMO [140,141].

Rituximab has been found to be clinically effective in NMO [33,34] and reduces the levels of anti-NMO IgG, but the antibody does not completely disappear [34,103,142]. There may also be a BAFF related transient elevation in the anti-NMO titres observed 2 weeks after the first injection of rituximab [142]. One possible explanation for this is that plasma cells, which do not express CD20 (the target of rituximab), may continue to produce antibody for a period of time, possibly at an increased rate due to the depletion of memory B cells and other modulatory Bcells (regulatory B cells) which do carry CD20 [143]. Repeated rituximab therapy may reduce the level of NMO IgG after the depletion of memory B cells and natural death of short-lived plasmablasts/plasma cells. Long-lived plasma cells may continue to secrete autoantibody despite the treatment with rituximab [144]. An alternative explanation is that it is purely through the modulatory effects of B cells on T cells that rituximab is having an effect, such that removal of memory B cells and activated B cells diminishes the proinflammatory milieu.

Disruption of the BBB is required for NMO IgG to enter the CNS. Serum from NMO patients can cause breakdown of the BBB [145,146] by an unknown mechanism. However, autoantibodies against cultured human brain microvascular endothelial cells were detected in 10 out of 14 seropositive NMO patients. These autoantibodies, which are distinct from anti-AQP4, were observed to reduce the expression of the tight junction proteins, that constitute the BBB, possibly through the autocrine secretion of vascular endothelial growth factor [146]. The BBB is further disrupted by injury to astrocytic foot processes sustained in the disease process [111].

**Figure 2:**
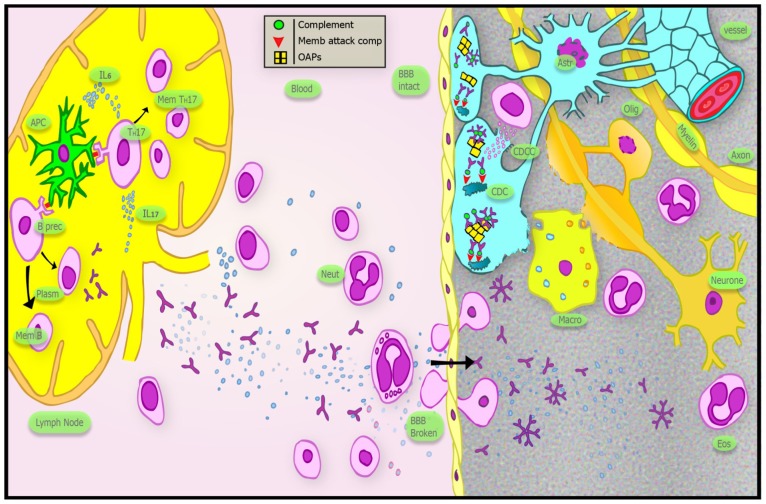
Schematic diagram of the immunopathogenesis of NMO. APC: antigen presenting cell; B prec: precursor B cell; Plasm: Plasma cell; Mem B: memory B cell; Mem Th17: memory Th17 cell; Neut: neutrophil; BBB: blood brain barrier; CDC: complement dependant cytotoxicity; CDCC: complement dependant cell mediated cytotoxicity; Astr: astrocyte; Olig: oligodendrocyte; Macro: macrophage; Eos: eosinophil.

Oligodendrocyte damage appears to follow immune mediated astrocyte injury [36,101] and these cells are not affected in *AQP4* null mice when exposed to NMO IgG and complement factors [100]. Glutamate induced excitotoxicity is one potential mechanism of oligodendrocyte injury. Glutamate is removed by excitatory amino acid transporter 2 (EAAT2) present on astrocytes in association with *AQP4*. In AQP4 transfected cells, the glutamate transporter is internalised with antibody-AQP4 complex, implicating glutamate excitotoxicity in NMO [50,51]. However, studies utilising primary astrocyte cultures as a substrate and *in vivo* studies have failed to demonstrate significant AQP4 complex or glutamate transporter internalisation [52,112]. Recent work has suggested that NMO IgG bound to the M23 isoform of AQP4 increases resistance to internalisation but does cause internalisation of the M1 isoform [112]. AQP4 has been observed in autopsy studies to be lost both in MS and NMO lesions [37,147]. This may be due to loss of astrocytes rather than internalisation. Loss of *AQP4* in EAE models has been observed to be neuroprotective [98], but it is likely that this effect is mediated by its water channelling action, which is not affected by NMO IgG binding [113,114]. Thus, loss of *AQP4* in NMO, either by internalisation or astrocyte loss, is one of the markers of the disease but may not be a primary pathogenic process. Whilst it is possible that the inflammatory process primarily targeting astrocytes causes bystander injury to nearby oligodendrocytes, widespread loss of oligodendrocytes is reported in early NMO lesions in the absence of a significant macrophage infiltrate [36]. The immunoglobulin predominantly seen in lesions of NMO patients in one study was of IgM type [148] but as noted earlier it is only detected in the blood of 10% of patients [62]. A possible explanation for this is intrathecal synthesis by local B-cells undergoing class switching. However, another study also showed IgG along with IgM in lesions of NMO patients thus supporting the role of IgG as a pathogenic antibody [36]. The immunopathogenesis of NMO is summarised in Figure 2.

### 2.7. Histopathology of NMO

**Figure 3:**
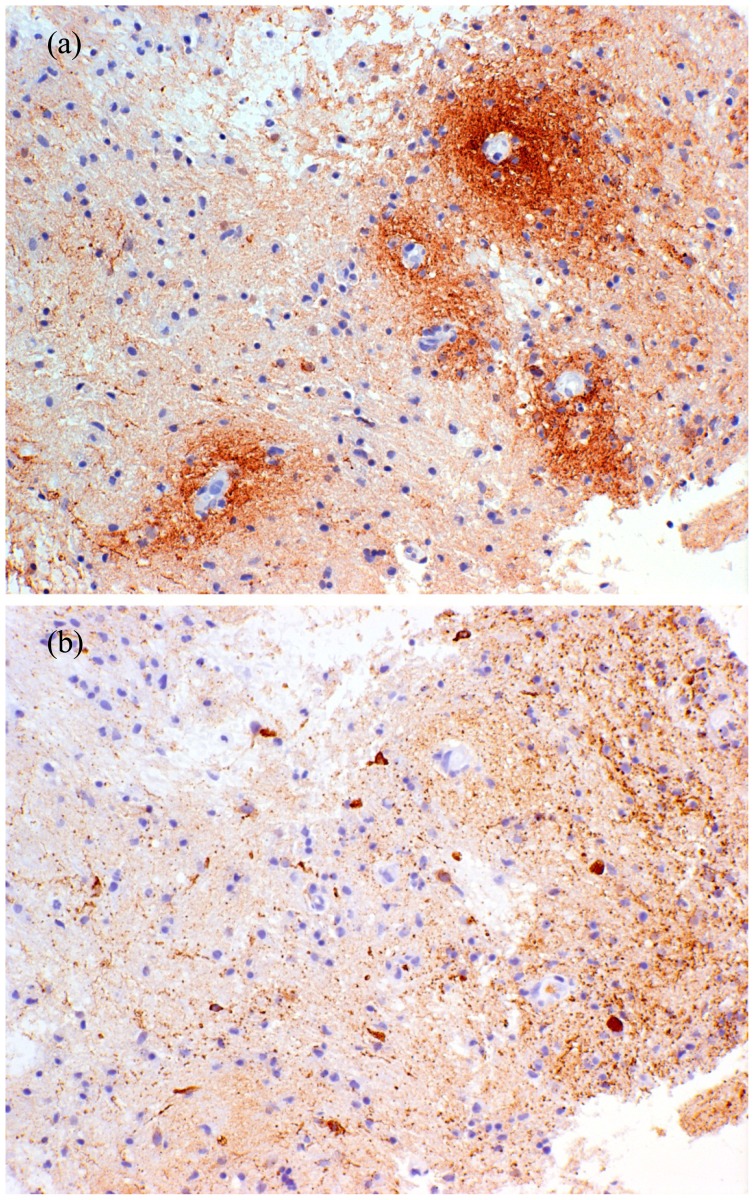
Serial sections from a biopsy of an early brain white matter NMO lesion immunostained for (**a**) C3d and (**b**) GFAP. (**a**) Typical perivascular complement deposition around numerous small vessels; **(b**) Particulate GFAP-positive debris with a perivascular accentuation indicating massive astrocyte destruction in a region with relatively preserved myelin architecture. Note that no normal astrocytes are visible.

The earliest pathological descriptions of NMO highlighted the frequent occurrence of necrosis with cavitation, hyalinisation of small vessels and perivascular inflammatory infiltrates. These features were helpful in distinguishing NMO from MS. More recent work has indicated the importance of perivascular deposition of immunoglobulin and complement [148] and its localisation to the vascular glial limiting membrane [149]. The infiltrate consists of macrophages, granulocytes and eosinophils with a general paucity of lymphocytes [122]. Modern histopathological techniques have demonstrated the specificity of acute fragmentation and loss of perivascular GFAP-positive astrocyte foot processes and their cell bodies as an early and consistent feature of NMO [36,150]. Whilst loss of *AQP4* is seen in NMO lesions [37,147], this is not consistent and is also seen in MS [151,152]. Relative preservation of myelin in early lesions with massive astrocyte destruction implicates astrocyte injury as the primary pathological event in NMO [37,147]. The presence of myelin loss and phagocytosis by macrophages in subacute NMO lesions suggests that this is a secondary phenomenon. Morphologically distinct unipolar and bipolar GFAP-positive astrocytes are seen in demyelinated subacute lesions suggesting that astrocyte progenitor cells may be responsible for reparative processes in NMO [37,147,153]. Interestingly, significant necrosis of axons was not seen in early and subacute lesions [36]. Intramyelenic oedema with partial astrocyte preservation may be responsible for the presumed vasogenic oedema seen on MRI as extensive white matter lesions in the brain [112]. The typical histopathological appearances in NMO are illustrated in Figure 3.

## 3. Conclusions

Great strides have been made in elucidating the molecular underpinnings of NMO, but aspects of the pathogenesis remain unknown and no currently proposed disease model is completely satisfactory.

It seems likely that NMO is an autoimmune disease that develops in genetically susceptible individuals. There is already some evidence for specific HLA alleles and polymorphisms of the *AQP4* gene being associated with NMO. Infection, vaccination, and neoplasm may trigger the disease process in some cases but a recognisable initiating event is lacking in most. In the schema presented in Figure 2, this trigger induces the release of inflammatory cytokines, including IL-6, and Th17 cells are stimulated. A subset of B-cells in the peripheral tissues is stimulated to produce anti-AQP4 IgG antibody. The BBB is made permeable to the autoantibody by either a systemic inflammatory response or a local pathological process in the CNS. Auto-reactive B cells may also enter the CNS and undergo class switching to produce IgM autoantibody. The antibody fixes complement on AQP4 expressing astrocytic foot processes. Stellate astrocytes are damaged by complement dependent cytotoxicity and complement dependent cellular cytotoxicity. Oligodendrocytes that are in close proximity to astrocyte foot processes are possibly affected through bystander inflammatory damage or, more likely, loss of trophic support from astrocytes. The resulting demyelination causes loss of saltatory conduction and conduction block, leading to neurological deficit. With continuing or repeated inflammation, substantial neuro-axonal loss ensues. The near absence of progressive forms of NMO (primary or secondary) suggests that neuro-axonal damage is the result of the acute inflammation, whether directly or indirectly, rather than as a result of a secondary degenerative process as appears to be the case in some forms of MS.

Some CNS sites, such as cerebellum, express *AQP4* abundantly but are not usually involved in NMO. Similarly, cerebral cortex expresses *AQP4* but is not affected. This may be due to the specific local immune environment. Elucidation of such protective mechanisms will be helpful in identifying new therapeutic targets. The processes responsible for oligodendrocyte loss are also largely unknown and the mechanism of axonal injury in chronic lesions is also poorly understood. Important questions regarding the aetiopathogenesis of seronegative cases with NMO phenotypes remain. Do these cases have a different antigenic target (heterogeneity) or do they represent a milder form of the disease (*forme fruste*), or do they reflect technical problems with the currently available NMO IgG assays (false negatives)?

As the first autoimmune disease of the CNS for which a specific autoantibody and antigenic target have been identified NMO represents a significant opportunity to better understand and hopefully treat its more heterogeneous and complex cousin, MS. Very recent research has highlighted a novel potential therapeutic approach utilising small-molecule inhibitors to reduce AQP4 mediated astrocyte cytotoxicity by disrupting the interaction between NMO-IgG and M23-AQP4 [154]. These experiments demonstrate the very direct way in which a greater understanding of the pathogenesis of autoimmune diseases such as NMO can lead to new therapeutic interventions. The prominent role played by IL-6 in NMO has led to the suggestion that IL-6 receptor blockade may be an effective therapy [72]. The development of fully humanised monoclonal antibodies against the B-cell antigens CD20 [155] and CD19 [156] raise exciting prospects for improved tolerability and potentially greater efficacy in the treatment of NMO.

## Figures and Tables

**Table 1:** Comparison of neuromyelitis optica (NMO) with multiple sclerosis (MS).

Clinical feature	NMO	MS
**Optic neuritis**	+++	++
Severe, bilateral	+++	(+)
**Myelitis**	+++	++
Partial	(+)	+++
Extensive (>3 segments)	+++	−
**CSF analysis**		
Oligoclonal bands	(+)	++
Elevated protein	+	(+)
Pleiocytosis	++	+
Lymphocytes	++	−
**MRI brain**		
Normal at onset	++	+
Hypothalamic/thalamic	+	−
Large hemispheric	+	+
Brainstem	+	+
“Barkhof” abnormal MRI	+	+++
**NMO IgG**	++	(−)
**Gender ratio (F:M)**	9:1	3:1

−: never seen; (+): can be seen but rare; +: sometimes seen; ++: often seen; +++: essentially universal.

**Table 2:** Human leukocyte antigen (HLA) associations in NMO and MS.

Disease	Population	N	DRB1	DQA1	DQB1	DPB1
**NMO**	Japan [81]	38				0501
	France [79]	45	03	-	02	
	Brazil [80]	54	03			
	USA [85]	154	(not 1501)			
	Han Chinese [82]					0501
	Martinque [86]	42	03		-	
**MS**	Australia [87]	100	1501	0102	0602	-
